# Computational Methods for Predicting Functions at the mRNA Isoform Level

**DOI:** 10.3390/ijms21165686

**Published:** 2020-08-08

**Authors:** Sambit K. Mishra, Viraj Muthye, Gaurav Kandoi

**Affiliations:** Bioinformatics and Computational Biology Program, Iowa State University, Ames, IA 50011, USA; skmishra@iastate.edu (S.K.M.); vrmuthye@iastate.edu (V.M.)

**Keywords:** alternative splicing, RNA-seq, machine learning, deep learning, recommender systems, multiple instance learning, mRNA isoforms, gene ontology

## Abstract

Multiple mRNA isoforms of the same gene are produced via alternative splicing, a biological mechanism that regulates protein diversity while maintaining genome size. Alternatively spliced mRNA isoforms of the same gene may sometimes have very similar sequence, but they can have significantly diverse effects on cellular function and regulation. The products of alternative splicing have important and diverse functional roles, such as response to environmental stress, regulation of gene expression, human heritable, and plant diseases. The mRNA isoforms of the same gene can have dramatically different functions. Despite the functional importance of mRNA isoforms, very little has been done to annotate their functions. The recent years have however seen the development of several computational methods aimed at predicting mRNA isoform level biological functions. These methods use a wide array of proteo-genomic data to develop machine learning-based mRNA isoform function prediction tools. In this review, we discuss the computational methods developed for predicting the biological function at the individual mRNA isoform level.

## 1. Introduction

**Figure 1:**
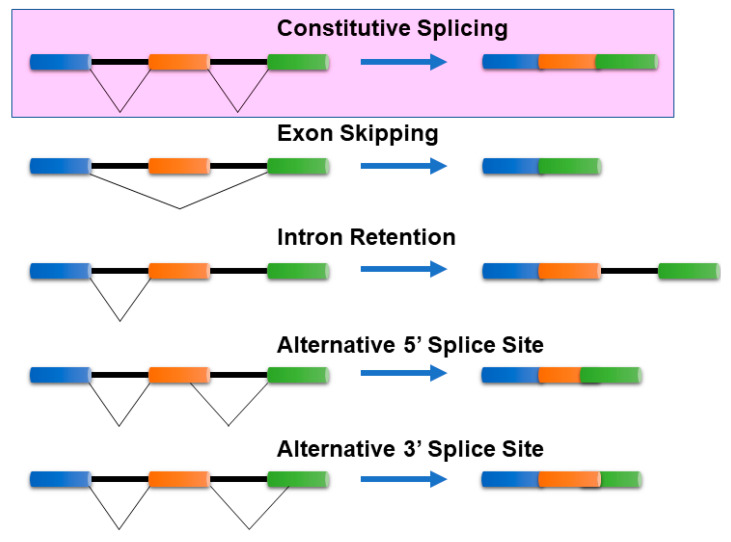
Common Alternative splicing events. The mechanisms of the most common alternative splicing events: Exon Skipping, Intron Retention, Alternative 5′ splice site selection, and Alternative 3′ Splice Site selection, are presented. There are several other alternative splicing events that are not shown here. The colored bars (blue, orange, and green) represent exons, while the black lines connecting them represent intronic segments. Exon skipping—The most prevalent mechanism in vertebrates and invertebrates where specific exons in the pre-mRNA are skipped in the mature mRNA transcript. Intron retention—Common in lower metazoans and plants, it is a process where an intron is retained in the mature mRNA, and Alternative 3′/5′ acceptor/donor sites—A process that involves exons that are flanked by competing splice sites on one end (3′/5′) and a fixed splice site on the opposite end, resulting in an alternative region that is either included or excluded in the mature mRNA.

Cells can produce multiple mRNA isoforms from a single gene because of a post-transcriptional regulatory mechanism, known as alternative splicing (AS). mRNA isoform sequences from a single gene may differ in a few base pairs up to several exons/introns. Such differences in the sequences can manifest either in the coding region or in the untranslated regions (5′ or 3′) of mRNA isoforms and can be characterized by several splicing mechanisms. In the absence of alternative splicing, all the introns are excised from the mRNA isoform. The commonly known AS mechanisms [1,2] include (Figure 1):

AS is a natural phenomenon and its prevalence is high in eukaryotes, such as mammals and plants [3]. The extent to which the eukaryotic genome undergoes AS is highly variable. Almost 61% intron-containing genes in *Arabidopsis thaliana*, 60% multi-exon genes in *Drosophila melanogaster* and as much as 90% multi-exon genes in human are alternatively spliced [4,5]. Interestingly, in the fission yeast *Schizosaccharomyces pombe*, only 2–3% of the genes undergo AS [6,7]. Despite that the alternative splicing in this organism also represents the important contribution for generating novel gene structures. Alternatively spliced mRNA isoforms from a single gene can have different functions and are characterized by their encoded protein isoforms [8]. Not all mRNA isoforms generated by AS are functional. Some isoforms differ in their biological properties, such as their catalytic activities, interactions of the mRNA isoform encoded proteins with other proteins and the sub-cellular localization of their encoded proteins [9]. AS is responsible for several functions within the organism including regulation of gene expression, response to stress, mRNA stability and protein diversity. The skipping of exon 63 of *SMG1* gene in peripheral leukocytes because of examination stress in male medical students is a remarkable example of AS [10]. Other interesting examples of AS include the genes *CASP3*, *MCL1*, and *BCL2* which produce mRNA isoforms performing completely opposite functions [11,12,13]. While several important cellular mechanisms and functions are attributed to AS, the underlying knowledge of how exactly splicing regulates such events is still unclear [14].

Due to the recent developments in massively parallel sequencing methods, a rapid collection of mRNA isoform level sequence and expression data has been generated. This wealth of data at the mRNA isoform level provides evidence confirming the differential expression of mRNA isoforms under different conditions [15,16,17]. Such evidence has led to the refinement and improvement in genome annotations by identifying new functions of genes attributed to an alternatively spliced mRNA isoform product which were previously unknown [14].

Most experiments that aim to characterize gene functions are typically performed at a gene level, i.e., the functional characterization is targeted only towards a given gene of interest and not specifically towards its mRNA isoforms. Such experiments do not account for the fact that a gene is a collection of multiple mRNA isoforms. Databases such as Gene Ontology (GO) [18], Uniprot Gene Ontology Annotations (Uniprot-GOA) [19], and Kyoto Encyclopedia of Genes and Genomes (KEGG) [20] that curate functional data are focused primarily on the canonical mRNA isoforms and do not have specific information on the functions of alternatively spliced mRNA isoforms. This dearth of gene function characterization at the mRNA isoform level has led to only a few hundred functional annotations for mRNA isoform functions in functional databases like GO. While most experiments aimed at annotating gene functions have lagged in describing and differentiating the functions of the mRNA isoforms of a given gene, numerous computational methods using machine learning (ML) and recommendation systems have been developed to annotate gene functions at the level of mRNA isoforms.

Previously, machine learning methods have been used to address a multitude of problems, some of which include, drug target discovery, gene function prediction, protein–protein interaction (PPI) prediction, protein structure and functional site prediction, and subcellular localization protein prediction [21,22,23,24,25,26,27]. More recently, several machine learning and recommendation system methods have also been developed to predict the biological functions of mRNA isoforms [28,29,30,31,32,33,34,35,36,37,38]. These methods have been successful in predicting gene functions at the level of mRNA isoforms and provide an added advantage over experimental approaches in terms of time and resources.

The problem of mRNA isoform function prediction is a challenging one. Many mRNA isoforms are potentially non-functional or are less important [39] and therefore introduce significant noise to any given dataset. Some mRNA isoforms are condition or tissue specific and thus, are functionally active only under specific conditions. The methods developed for predicting gene function cannot be directly used for mRNA isoform function prediction because these methods ignore the distinct functions of alternatively spliced mRNA isoforms of the same gene. Despite these limitations, the recently developed computational methods have been able to identify mRNA isoforms that are strongly associated with a gene’s annotated functions, differentiate functional mRNA isoforms from non-functional ones, and even predict the functions of novel mRNA isoforms.

mRNA isoform function prediction methods based on machine learning and recommendation systems typically involve the following steps:

Training label generation—Involves creating a training dataset and assigning labels to the data points. An example of label assignment in the context of mRNA isoform function prediction could be assigning “functional” and “non-functional” labels to all mRNA isoforms in the training data.

Feature calculation—Involves calculations of features for all mRNA isoforms in the training data. Depending on the model, the features may be calculated for each mRNA isoform, mRNA isoform pairs or genes.

Training the model—An initial model is trained on the training data

Model evaluation—The predictive performance of the trained model is evaluated on the training data, often using different cross validation techniques.

Feature and model parameter optimizations—The parameters of the trained model and the input mRNA isoform features are further optimized to improve prediction performance.

Prediction on test dataset—The optimized model is evaluated on a test dataset that is independent of the training data.

**Figure 2:**
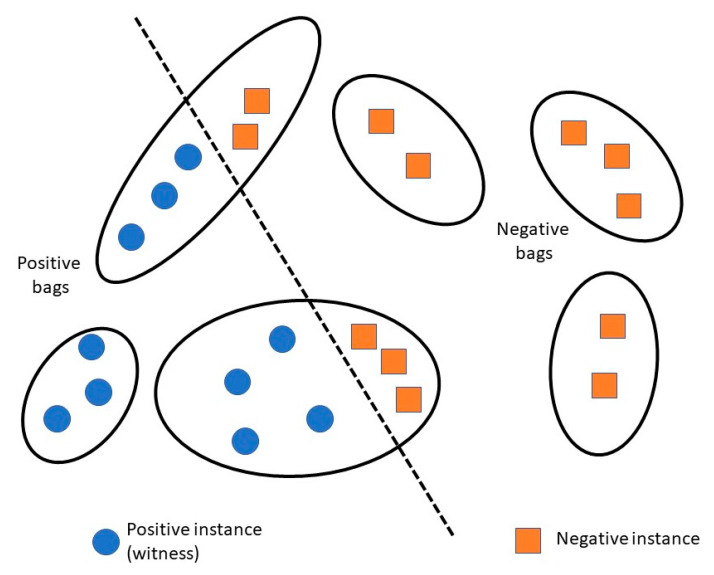
Overview of multiple instance learning (MIL) framework. Each gene (black ellipse) is considered a bag, where each mRNA isoform (circle or square within the ellipse) is considered an instance of the bag. A gene associated with a function is a positive bag, and all instances (mRNA isoforms) associated with that function are called “witnesses”.

Few mRNA isoform function prediction methods use more traditional and widely used algorithms like logistic regression and random forest, while some others use deep learning or recommendation systems. However, a lot of the existing methods use the multiple instance learning (MIL) framework, with a machine learning algorithm as a base-learner (Figure 2). In MIL, a gene is considered a “bag” and an mRNA isoform is considered an “instance” of the bag. A gene associated with the function under study is referred to as a “positive bag” and the mRNA isoforms of the gene responsible for the gene’s function are known as “witness(es)”. There is at least one witness in a positive bag. The aim is to identify a subset of mRNA isoform witnesses from a positive bag that maximize their difference from the negative mRNA isoforms.

This review focuses on the machine learning and recommendation system approaches for mRNA isoform function prediction. We also consider MIL-based methods since they use machine learning algorithms as a base-learner within the MIL framework. This review is organized into the following sections: 1. mRNA isoform level functional network prediction methods; 2. MIL-based mRNA isoform function prediction methods; 3. Deep learning-based mRNA isoform function prediction methods; and 4. Recommender system-based mRNA isoform function prediction methods. A glossary of frequently used terms in the field are provided in Table 1. Finally, we discuss ways that can help further refine annotations at the mRNA isoform level.

## 2. mRNA Isoform Level Network-Based Methods

Fundamental cellular processes are regulated and performed by protein–protein interactions (PPIs). Identifying the interacting partners of a protein can help us better understand its different roles within and beyond the cell. In PPIs, the term “protein” generally refers to “protein variants encoded by all mRNA isoforms of the gene”. PPIs do not take into consideration the mRNA isoforms from which the proteins are encoded and thus, lack a one-to-one mapping between mRNA and protein isoform data. It is widely acknowledged that AS can result in altered protein domains and structures, thereby modulating PPIs. These differences in the interaction behavior can lead to loss or gain of functional partners that could be key links of pathways [40]. It is therefore likely that each protein isoform encoded by an mRNA isoform of a gene, interacts with multiple different partner proteins many of which might be mutually exclusive. Such an ensemble of isoform-specific interactions could regulate different functions. Pipelines for predicting gene function using gene level networks cannot be directly employed for solving the problem at the mRNA isoform level. This is mainly because most functional data at the genomic level is analyzed for genes and not mRNA isoforms. Because of this there are very few mRNA isoform pairs with functional information for developing models for mRNA III functional network. In this section we discuss methods developed to predict mRNA isoform–isoform interaction (IIIs) functional networks. Unlike co-expression networks where an edge between two mRNA isoforms means that they have similar expression patterns, in a functional network, an edge indicates that the two mRNA isoforms are involved in the same function.

### 2.1. Isoform–Isoform Interaction Database (IIIDB)

In a two-step procedure, Tseng et al. developed IIIDB [33], an mRNA isoform level functional network for humans. The authors first predicted IIIs at the mRNA isoform level and then identified functional modules in the IIIs network. The IIIDB is developed using a series of RNA-Seq datasets along with domain–domain interactions. A set of 19 RNA-Seq datasets with at least 10 samples in each dataset from different physiological and experimental conditions was used. All human mRNA sequences with a protein sequence in NCBI RefSeq (31,454 sequences; Jan 2013 version) were used as the transcriptome annotations. The protein expression level was assumed to be the same as that of the mRNA isoform expression level. If multiple mRNA isoforms corresponded to the same protein sequence, the protein expression was calculated as the sum of all the corresponding mRNA isoform expression levels.

mRNA isoform level co-expression networks were constructed for each RNA-Seq dataset by calculating the Pearson correlation coefficient (PCC) between the expression of all mRNA isoform pairs in that dataset. The PCCs were converted to z-scores and the z-scores were standardized to have zero mean and unit variance to normalize the raw PCC values. A logistic regression model was then used to predict if there exists an edge between any given mRNA isoform pair.

The explanatory variables used in the logistic regression model included the correlation values from 19 RNA-Seq datasets and the domain–domain interaction scores from DOMINE database [41]. For training and evaluating the logistic regression model, the authors selected the positive set as: 1. mRNA isoform specific PPI from IntAct and 2. PPI involving genes producing single mRNA isoforms only. To construct the negative set, the authors selected those mRNA isoform pairs for which one mRNA isoform is predicted to be a plasma membrane cellular component, while the other mRNA isoform is predicted to be a nuclear cellular component. The authors used MODES network clustering method [42] for functional mRNA isoform module discovery.

The authors verified the performance of their approach on the mRNA isoforms of APP gene, which is associated with Alzheimer’s disease. They observed their method to have correctly predicted the mRNA isoforms that were reportedly associated with Alzheimer’s. A major drawback of IIIDB is that it limits its prediction to PPIs existing in IntAct. Because of this, new functional annotations cannot be assigned at the gene or at the mRNA isoform level. Tissue, cell and/or condition specific interactions and functions are ignored. The selection of negative set based on predicted subcellular locations, while better than selecting random non-positive set, is still biased and propagates “error of prediction”.

### 2.2. Network of Splice Isoforms for Mouse

The authors applied a Bayesian network-based MIL approach to address the problem of mRNA IIIs functional network prediction [35]. Using this MIL approach, the authors built an mRNA isoform-level functional relationship network of mouse by integrating data from multiple sources.

In this approach, a pair of functionally related genes (positive bags) was assumed to contain at least one pair of functionally related mRNA isoform pair. Additionally, in a pair of functionally unrelated genes (negative bags), none of the mRNA isoform pairs were assumed to be related functionally. Through this approach, the final goal was to recognize functionally related mRNA isoform pairs from positive bags. A naïve Bayesian network was used as the base-learner for the MIL framework. The authors developed a “single-instance bag MIL” algorithm, which improves on the performance of previous MIL approaches and reduces the false positive rate.

The authors incorporated data from multiple sources. This included 65 heterogenous datasets: RNA-seq, exon array, pseudo-amino acid, and isoform-docking data. For the first three dataset types (RNA-seq, exon array, and pseudo-amino acid) the feature input was the correlation between each mRNA isoform pair. For the isoform-docking data, the docking score between two proteins was the input feature. Five-fold cross-validation was used to evaluate the performance of the four types of input features. The most discriminative among the four feature types was the isoform-docking score. The best performance was achieved by using an integrated network (using all dataset types), compared to individual dataset types.

Functionally related gene pairs were defined as two genes which were assigned the same GO biological process term or pathway. 675,124 positive gene pairs were identified using data from GO, KEGG, and BioCyc [11] databases. Negative pairs, i.e., functionally unrelated genes, were chosen randomly. The RefSeq gene build version 37.2 of mouse was used to build the mRNA isoform network.

This method was tested using multiple approaches. The first approach was using simulated datasets from the RefSeq gene build. They focused on two parameters: 1. The mean difference (MD) of the values between functionally related and functionally unrelated mRNA isoform pairs, and 2. the ratio of multi-isoform genes to the total number of genes (MGR ratio). The approach was shown to work well with genomic data of very weak MD, varying MGR ratios, as well as different combinations of both MD and MGR.

The model was validated using single mRNA isoform gene pairs, that was referred to as the “gold standard dataset”, using cross-validation. The approach was shown to be accurate when the Area Under the Receiver Operating Curve (AUROC) values (0.656) were analyzed. Furthermore, using data from the Corominas database, a database of experimentally verified isoform–isoform interactions [43], the authors showed that their approach was able to identify which mRNA isoforms were interacting. Their mRNA isoform-level functional relationship network identified varying functions of different mRNA isoforms from the same gene in several cases. For example, they looked at the local networks of the mRNA isoforms of the Anxa6 gene that are known to have two different functions. Only 13 out of 25 neighboring mRNA isoforms were shared in their local networks.

The authors created an mRNA isoform-level functional relationship network of mouse, using data from multiple sources, and a MIL approach. They rigorously tested their approach using both simulated and experimentally verified data. However, they used a 2-class classification system, i.e., a gene was either positive or negative for a GO term. Furthermore, in their approach, negative gene pairs were randomly selected, thereby possibly introducing bias in their model. The GO hierarchy was not used for generating the training labels, thereby resulting in the loss of important functional information. Covariates such as tissue, condition, age, or sex specificity were not considered.

### 2.3. Tissue-spEcific mrNa iSoform functIOnal Networks (TENSION)

In a different approach, the authors formulate the problem of mRNA III functional network prediction as a simple supervised classification problem (edge prediction) and generate tissue level mouse mRNA III functional networks [29]. The 17 tissue-specific mRNA III functional networks for mouse were developed using a series of RNA-Seq datasets, mRNA sequence-based properties, and protein sequence features. The NCBI *Mus musculus* genome assembly (GRCm38.p4) was used and all mRNA isoforms with available protein sequence (longer than 30 amino acids and no non-standard amino acids) were used. For tissue-specific mRNA III functional networks, only those tissues were included for which there were more than 10 samples.

These filtering criteria resulted in the selection of about 76,000 mRNA isoforms from approximately 22,000 mouse genes. A total of 359 RNA-Seq samples from around 20 tissues (17 tissues have more than 10 samples) were processed to calculate the log-transformed FPKM values for every mRNA isoform. For every tissue that had more than 10 samples, a PCC value was obtained for every mRNA isoform pair. This resulted in 18 PCC values for all mRNA isoform pairs (an additional value was obtained by using expression from all 359 samples) and were used as features along with those obtained from the mRNA and protein sequences. Four PCC values were obtained from the mRNA isoform sequences and another five from the protein sequences. All PCC values were transformed using Fisher’s z-transformation. Finally, 27 mRNA isoform–isoform level features were obtained and used to train random forest models for predicting tissue-specific mRNA III functional networks.

The training and testing datasets were generated by combining the data from GO biological process annotations, KEGG pathways, BioCyc pathways and PPIs from multiple databases. GO annotations with little support such as those inferred from electronic annotations were not included in the generation of training and testing datasets. The annotations in the GO were propagated along the GO hierarchy (“true path rule”) to increase the number of positive annotations. GO terms with more than 10 genes and less than 1000 genes annotated were considered. Single mRNA isoform producing genes were used to generate the positive labels, while GO annotations tagged with a “NOT” qualifier were used for the negative labels.

If two genes produce a single mRNA respectively and are co-annotated to the same GO term, pathway, or PPI, the two mRNAs were assumed to be functionally related (positive label). If a gene is tagged with a “NOT” qualifier for a GO term, it was considered non-functional (negative) for the respective GO term. Because there are only few hundred such annotations in the GO, all such annotations are also propagated to the child terms of the respective GO term (inverse of “true path rule”). This increases the number of negative annotations significantly. All mRNA IIIs which involve at least one negative annotation are considered as functionally unrelated (negative label). All mRNA IIIs labelled as both positive and negative were considered as positives.

The authors generated a reference mRNA III functional network by using all 27 features and 17 tissue-specific networks by removing the tissue-specific RNA-Seq expression-based feature. mRNA IIIs were considered tissue-specific functional pairs if the prediction for the mRNA IIIs changed from positive to negative after removing the tissue-specific RNA-Seq feature. Similarly, mRNA IIIs were considered tissue-specific non-functional pairs if the prediction changed from negative to positive. The predictions were evaluated and validated by randomized positive and negative class labels, updated GO annotations, stratified 10-fold cross-validations, and by literature evidence. Additional validation was also performed by using tissue-preferred genes from the transcriptomic BodyMap of mouse and 20 ubiquitously expressed genes [44,45]. The authors obtained significant improvement (20 points) in performance compared to a previous method [35].

Although the authors use a more robust strategy to define their positive and negative labels, there are still a few shortcomings of the proposed framework. Like previous studies, co-variates such as age, sex and developmental stages were not considered. Instead, generic organism (or tissue) level mRNA III functional networks were built. While several validation approaches have been used to validate the predictions, no experiments were performed to validate novel predictions. TENSION is readily available through Figshare (https://doi.org/10.25380/iastate.c.4275191) [46].

## 3. mRNA Isoform Level Machine Learning Methods

### 3.1. Multiple Instance Learning (MIL)

The MIL framework has been used in several mRNA isoform function prediction tools. Here, we review four MIL-based isoform function prediction methods.

#### 3.1.1. IsoPred (mi-SVM)

Eksi et al. [38] developed a MIL approach, IsoPred, using only RNA-seq data from mouse. isoPred follows the conventions of the MIL approach as discussed earlier (see Introduction, Figure 2). The objective here is to identify the mRNA isoform “responsible” for the gene’s function, therefore generating functional annotations at the mRNA isoform level.

The authors trained a support vector machine (SVM) model coupled within the MIL framework (mi-SVM) on mouse RNA-seq data to predict the functions of the mRNA isoforms. The dataset comprised RNA-seq data from 365 RNA-seq experiments from mouse and included 19,209 genes and 24,274 mRNA isoforms. These RNA-seq datasets come from a wide variety of tissue and experimental conditions. The annotations were taken from the GO database and all genes associated with a given GO term or its descendants were treated as positives, while the rest were considered negative. Bootstrap bagging was applied to obtain final mRNA isoform level scores.

The authors performed three types of tests to evaluate the predictive performance of their method. First, they performed 5-fold cross-validation of gene-level predictions using single and multiple mRNA isoform genes. Their tests revealed that their method shows superior performance for multi-mRNA isoform genes (AUROC: 0.71), when compared to single mRNA isoform genes (AUROC: 0.65). This suggests that their method can assign the function of a gene to at least one mRNA isoform. Next, they performed validation of the predicted functional mRNA isoforms using splice variant protein expression data in normal mammary tissue. They observed strong correlation between the mRNA isoform predicted to have higher score and its protein expression. Finally, they analyzed the disparate functions predicted for the mRNA isoforms of *CDKN2a* and *ANXA6* genes by comparing the predicted protein structures of the mRNA isoforms. They found that the different functions predicted for the mRNA isoforms could be explained by the differences in the protein structures.

Only expression profile of mRNA isoforms was used by IsoPred to characterize them. Although RNA-Seq samples have been used from multiple different tissue and experimental conditions, no tissue-specific predictions are made. No distinction is made between the covariates such as age and sex. Random unannotated genes were used as negative sets which can introduce bias in the training and testing dataset.

#### 3.1.2. Instance-Oriented Multiple Instance Label Propagation (iMILP)

The overall idea of instance-oriented Multiple Instance Label Propagation (iMILP) [37] is to model a gene-interaction network describing co-expression, which is derived from RNA-Seq data. Such a model has “bags” (genes) of “nodes” (instances) where, each node is represented by a mRNA isoform of a gene and each bag is assigned a label corresponding to the gene’s function. The algorithm iterates over multiple mRNA isoform networks, each network created from a different RNA-seq dataset, updating the labels in each iteration and ultimately, identifying mRNA isoforms of a gene carrying out a certain function.

iMILP is composed of two modules—(1) network selection and combination, and (2) mRNA isoform function prediction. For each GO term, the first module chooses an optimal subset of all input mRNA isoform co-expression networks and combines them into a single network. In this step, each individual network is treated as a “feature”, and the problem of selecting optimal networks is treated as a “feature selection problem”. The wrapper method of feature selection, that uses a greedy sequential forward strategy, is used to select optimal networks. The second module, the iMILP predictor, takes the output from the first module and predicts mRNA isoform-specific functions. The strategy used by the predictor for label propagation is that a node (mRNA isoform) from a positive bag (gene assigned to a particular GO term) that is linked to more nodes from positive bags is assigned a higher prediction score for that GO term. Conversely, a node from a positive bag that is not linked to any other node from positive bags receives a prediction score of zero. Using this strategy, the predictor identifies which instances in a positive bag are positive. One of the advantages of iMILP, over other MIL predictors, is that it uses a 3-class classification approach for each label, i.e., positive, negative, and unknown (mRNA isoforms of genes that have no annotated function).

The authors used 29 human RNA-Seq datasets, each having at least 6 experiments, to construct mRNA isoform co-expression networks for each dataset. To identify functional homologs, each mRNA isoform from a given RNA-Seq dataset was mapped on to 31,454 human mRNAs taken from NCBI RefSeq database. For each dataset, the co-expression network was created using only those mRNA isoforms that mapped on to the 31,454 human mRNAs. To construct an mRNA isoform network, the authors used PCC for mRNA isoform expression and then used the GO data as labels for functions. The PCC were transformed to z-scores and normalized to have zero mean and unit variance.

The method showed an average cross-validation AUROC score of 0.67 averaged across all GO terms. The authors also verified the prediction performance on different GO branches (biological process, cellular component, and molecular function) with different number of genes in each branch and observed an AUROC score > 0.6 in all cases. The authors then verified the performance of their method for human protein isoforms. Their method predicted 70,392 isoform-level functions, 13,621 of which were de novo predictions and 8856 predicted isoform functions had at least one annotation inherited from their host genes. In addition, their method exhibited high predictive accuracy for the functions of five mRNA isoforms of the tumor suppressor gene (*TP53*): p53α, p53β, p53γ, Δ40p53α, and Δ133p53α through the GO term “regulation of apoptotic process” or its descendants. Another 6 mRNA isoforms corresponding to the apoptosis regulatory genes: *BCL2L1*, *CFLAR* and *DNAJA3* were also used for validation. Each mRNA isoform is either involved in the positive or negative regulation of apoptosis and this method, correctly predicted the functions of 8/11 (72.7%) and 9/11 (81.8%) of the positive and negative mRNA isoform, respectively.

iMILP used only expression profile of mRNA isoforms to characterize them. While RNA-Seq samples have been used from multiple different tissue and experimental conditions, no tissue-specific predictions are made. Covariates such as age and sex were also not distinguished in the developed model. Genes annotated to sibling GO terms were used as negative sets which can introduce bias in the training and testing dataset.

#### 3.1.3. IsoFunc (MIL-SVM)

This method makes assumptions similar to that of Eksi et al. [38]—(1) of all the mRNA isoforms of a gene, at least one mRNA isoform is responsible for performing the gene’s verified function, and (2) none of the mRNA isoforms of negative genes are associated with that function. The underlying aim, similar to Eksi et al. [38], is to identify subsets of mRNA isoforms of positive genes that exhibit maximum difference between them and the mRNA isoforms of the negative genes. For achieving this aim, the authors used the “maximum-margin-based classification” approach. Their method implements MIL on a subset of mRNA isoforms maximizing their objective function using SVM.

The method uses 248 RNA-seq runs (127 samples) from human taken from the ENCODE [47] project. The authors then selected 11,946 genes with 59,297 mRNA isoforms that had protein-coding mRNA isoforms for their study. All genes annotated to a GO term (and its descendants) were considered as positive for the GO term under study. All remaining genes were considered as negative for the GO term under study.

The authors performed 5-fold cross-validation by creating positive and negative gene-sets for each GO term and observed a median AUROC of 0.64. The authors also divided the genes based on the number of protein-coding mRNA isoforms and observed performance improvement for genes with higher number of mRNA isoforms. The authors also validated their method on two genes: *ADAM15* and *LMNA/C* for which there are experimental functional evidence at the mRNA isoform level. *ADAM15* has been shown to have two mRNA isoforms—ADAM15A and ADAM15B and this method was able to distinguish between the functions of these mRNA isoforms. Another gene, *LMNA*, which is associated with three mRNA isoforms—lamin A, progerin, and lamin C, was also used for validation. The authors observed distinct GO terms for each mRNA isoform, which were consistent with previous literature.

Like several other mRNA isoform function predictors, this method suffers from the drawback of using a 2-class classification system for labelling genes, i.e., a gene can only be “positive” for a GO term or “negative”. Only mRNA expression profile has been used as an input to IsoFunc. No tissue or condition specific predictions are made. Covariates such as sex and age are also not considered. Random unannotated genes were used as negative for each GO term, which can introduce bias in the training and testing dataset.

#### 3.1.4. Weighted Logistic Regression-Based MIL method (WLRM)

The authors have developed a Weighted Logistic Regression-based MIL (WLRM) model that uses a “nonconvex sparsity-inducing regularizer” in the framework of MIL. A mapping of discriminative feature space of the original gene level feature is learned by sparse projections onto simplex. Several smooth and non-smooth loss functions like hinge loss and logistic loss can be incorporated in the proposed framework. An efficient block coordinate descent algorithm is also developed to solve the highly nontrivial non-smooth and non-convex optimization problem formulated in the WLRM framework.

Similar to IsoFunc [36], WLRM used a total of 248 human RNA-seq runs (127 samples) taken from the ENCODE [47] project. The authors performed filtering based on the percentage reads mapped to the reference human genome and average expression of transcripts. They arrived at a total of 11,946 genes with 59,297 mRNA isoforms. This set of genes was then annotated based on Gene Ontology. For a given GO term, genes (and their corresponding mRNA isoforms) that were annotated for this term were labelled as positives and the remaining set of genes and mRNA isoforms were treated as negatives. The authors used 94 benchmarked GO terms to validate their method.

The WLRM approach was validated using 5-fold cross-validation for each GO term and then compared with three existing methods—miSVM [36], miFV [48], and miVLAD [49] using cross-validation results. The performance comparisons were carried out for five different groups of the 94 GO terms, each group created based on the number of genes associated with each term. The authors observed that their method showed superior performance (median AUROC 0.691) for the GO terms associated with fewer genes compared to the three other methods. However, with an increase in GO term size, WLRM shows higher specificity and accuracy compared to the three methods. More importantly, this method also exhibits smaller execution time for the different sizes of GO terms compared to the other methods.

This method uses a 2-class classification system for their labels, where a gene can be either “positive” or “negative” for a particular GO term, which may introduce bias in their training and testing datasets. Unannotated genes were used as the negative set, which introduces bias in the training and testing dataset. Only expression profile has been used to characterize the mRNA isoforms. No tissue or condition specific predictions are made and covariates such as sex and age are also not considered.

### 3.2. Deep Learning Based Methods

**Figure 3:**
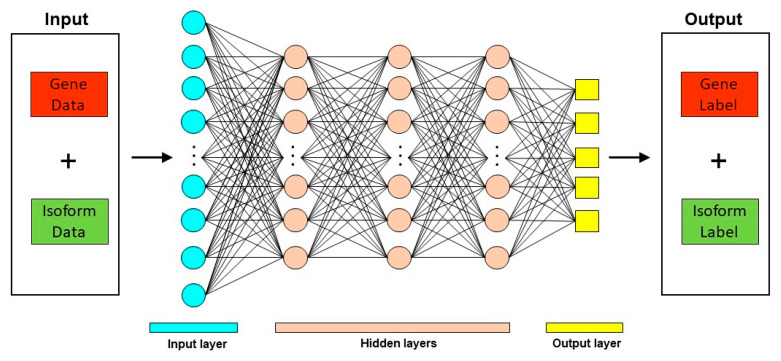
Deep Learning approach for mRNA isoform function prediction. In these methods, gene and mRNA isoform level features are used as input to a deep neural network which consists of multiple hidden layers. The output from these deep neural networks are the predicted gene and mRNA isoform level function predictions.

Deep learning techniques have been used in diverse biological contexts, like protein function prediction and prediction of protein subcellular localization. Here, we review two tools that employ a deep learning approach for predicting mRNA isoform specific functions—DIFFUSE and DeepIsoFun. A basic overview of the deep learning approach as applied to mRNA isoform function prediction is shown in Figure 3.

#### 3.2.1. DeepIsoFun

Previous methods have tried to address the issue of mRNA isoform function prediction by using a semi-supervised learning technique called MIL [30,36,37,38] However, the lack of labelled training data is reflected in their poor performance. To improve performance, DeepIsoFun [31] combines MIL with domain adaptation (DA) [50] to predict the functions of mRNA isoforms, using GO and RNA-Seq expression data.

The DeepIsoFun framework can be categorized into two domains: The gene domain and the mRNA isoform domain. In the mRNA isoform domain, as per MIL, each gene is considered a bag, with all its mRNA isoforms as instances in that bag. Additionally, individual genes have expression information, and are associated to functions in GO. Thus, by definition, a gene is both, a bag in the mRNA isoform domain, and an instance in the gene domain. The relationship between the function and expression of a gene can be transferred to the mRNA isoform domain, using the DA technique. The application of the DA technique, to generate labelled training data and transfer knowledge between the two domains, sets DeepIsoFun apart from the other MIL-based mRNA isoform function predictors. DeepIsoFun with DA significantly outperforms DeepIsoFun without DA.

The Deep Neural Network (DNN) architecture of DeepIsoFun is comprised of four modules: 1. An autoencoder consisting of two fully connected hidden layers to extract common features of both domains, and three classifiers implemented as parallel neural networks (NN), each consisting of one hidden layer, 2. Gene function predictor (labels the function of each gene), 3. mRNA isoform function predictor (labels the function of each mRNA isoform), and 4. A domain label predictor (ensures knowledge transfer from the gene domain to the mRNA isoform domain). The three classifiers and the autoencoder form a deep feed-forward network. These NNs are trained for each GO term. GO terms that are infrequent (associated with less than 5 proteins) and those inferred from electronic annotation were excluded in the cross-validation training. The performance of DeepIsoFun was shown to be robust across the three GO branches (Biological Process, Molecular Function, and Cellular Component).

The performance of DeepIsoFun was analyzed using datasets from phylogenetically distant organisms. First, three datasets were used from two organisms: *Homo sapiens* and *Mus musculus* to compare DeepIsoFun to three existing methods: 1. iMILP [37], 2. mi-SVM [36,38], and 3. WLRM [30]. In all the three datasets, DeepIsoFun outperformed the other predictors (Dataset #1 AUROC of 0.742 vs 0.64 (iMILP); Dataset #1 AUROC of 0.735 vs 0.679 (mi-SVM) and 0.69 (WLRM); Table 1 and Table 2 from [31]). Additionally, DeepIsoFun was compared to the other predictors on two more datasets- *Arabidopsis thaliana* and *Drosophila melanogaster*. DeepIsoFun outperformed the other predictors on these two datasets as well (Tables S2 and S3 from [31]). These comparisons were made using a small subset of 117 GO Slim terms which have been used in previous studies [51]. Validation was carried out using 18 human genes with multiple mRNA isoforms- some with pro-apoptosis and some with anti-apoptosis functions. Compared to the remaining predictors, DeepIsoFun was better at differentiating the anti- and pro-apoptosis functions of these mRNA isoforms.

In summary, due to the application of the DA technique, DeepIsoFun showed improved performance over the existing mRNA isoform function predictors. The code for DeepIsoFun has been made readily available on the project’s GitHub repository (https://github.com/dls03/DeepIsoFun/) along with instructions for installation and use. Despite the improved performance of DeepIsoFun, the lack of labelled training data for mRNA isoform function prediction and imbalanced GO data, leaves room for improvement. For instance, the comparison of DeepIsoFun with other methods showed the best AUROC and AUPRC as 0.7 and 0.3 respectively for DeepIsoFun. Additionally, DeepIsoFun uses a 2-class classification system, where an isoform can either be positive or negative with respect to each GO term, unlike a 3-class system that adds an “unknown” label. Thus, DeepIsoFun assigns a “negative” label to gene that is not assigned to a particular function, introducing bias in training and testing datasets. DeepIsoFun, like the other MIL-based methods, uses only expression information to assign function of a gene to its mRNA isoforms, which has been suggested to limit its performance. DeepIsoFun does not assign new functions at any of the two, gene or mRNA isoform levels. Tissue, condition, sex, and age specific functions of mRNA isoforms are ignored.

#### 3.2.2. Deep Learning-Based Prediction of IsoForm FUnctions from Sequences and Expression (DIFFUSE)

Several of the recently developed methods for functional annotation of mRNA isoforms use information from expression profiles alone. There is another possible source of information overlooked by these methods: The mRNA isoform sequences themselves. The mRNA isoform sequences can contain information like active sites, binding sites, signal peptides, motifs, and protein domains that can provide information about the function of that specific mRNA isoform. For instance, alternative splicing of mouse transcription factors changed domain composition of the mRNA isoforms, leading to tissue-specific isoforms with distinct functions [52]. Deep learning-based prediction of IsoForm FUnctions from Sequences and Expression (DIFFUSE) [32] uses both mRNA isoform sequence specific features and information from expression profiles to predict mRNA isoform functions.

DIFFUSE consists of two important modules: (1) A DNN that extracts features from mRNA isoform sequences and conserved domains, and (2) a conditional random field (CRF) that outputs a prediction by taking into account both the DNN score and co-expression information. The input to the DNN is comprised of trigrams generated from protein sequences and conserved domains. Here, it is important to note that a “domain” (protein domain) refers to structural and evolutionary building blocks of proteins. For each GO term, the DNN uses sequence information to measure how likely it is that the mRNA isoform in question is positive for that GO term. In the second module, the CRF, co-expression information is extracted. Due to the lack of mRNA isoform label information, a semi-supervised training algorithm is used to train the DNN and CRF together. The labels are updated iteratively through mean field approximation.

DIFFUSE was compared to DeepIsoFun, mi-SVM, iMILP, and WLRM, focusing on a small set of GO Slim terms, and three datasets. DIFFUSE showed a significant increase over the performance of DeepIsoFun, which itself was shown to outperform the remaining three predictors. An ablation study by removing the individual modules (CRF, conserved domain features, and sequence features) showed that information contained in the conserved domain features was important for predicting mRNA isoform function. Further, the authors analyzed correlation between predicted functions and mRNA isoform sequences, expression profiles and protein structures. They found that functional similarity was more correlated with sequence similarity than expression similarity. DIFFUSE was validated using information for 14 mRNA isoforms of 6 genes with strong functional evidence. DIFFUSE predicted the function accurately for 11 of them, more than the other methods.

DIFFUSE suffers from a similar limitation, as seen in DeepIsoFun. DIFFUSE, like DeepIsoFun, uses a 2-class classification system; a gene can be “positive” or “negative” with respect to a specific GO term. This assumes all unassigned gene-GO term relationships as negative which introduces bias in the training and testing data. Like previous methods, DIFFUSE fails to make any tissue, cell, or condition specific mRNA isoform function predictions. Unlike previous methods, DIFFUSE utilizes the protein sequence and domain information to improve the performance of mRNA isoform function prediction task, however, the mRNA isoform sequence is yet to be used as another source of features. DIFFUSE ignores mRNA isoforms which are not expressed in the expression datasets used in the study, which limits the method to a smaller subset of all human mRNA isoforms. The code of DIFFUSE has been made readily available in their GitHub repository (https://github.com/haochenucr/DIFFUSE).

### 3.3. Recommendation System-Based Methods

**Figure 4:**
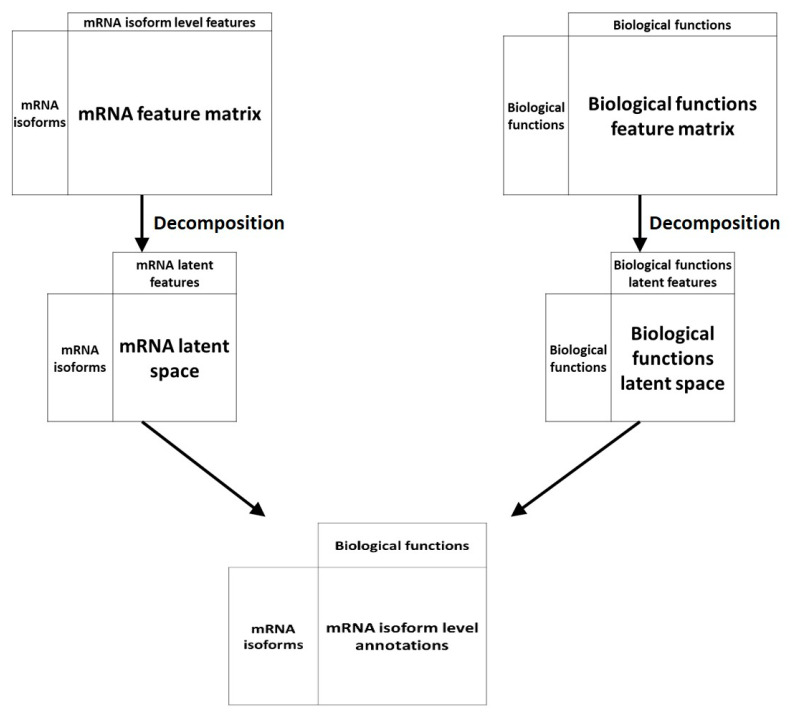
An overview of recommendation system approaches for mRNA isoform function prediction. In recommendation system-based approaches, the mRNA isoform level features and the features at the biological functions like GO are projected to a latent space which are then associated by a decomposition unit to produce the final mRNA isoform level function recommendations.

Another machine learning technique has been applied to the problem of mRNA isoform function prediction: Recommendation systems. Recommendation systems, used widely in non-biology fields like e-commerce and online advertisements, are a set of tools that can provide suggestions or recommendations of “items” that are useful to a “user”. In the context of biological questions, “users” are typically entities like genes or mRNA and “items” are a biological property like function (Figure 4). Below, we review two recommendation system-based mRNA isoform function predictors—mFRecSys and DisoFun.

#### 3.3.1. mFRecSys

A recently developed predictor, mFRecSys [34], uses existing information of mRNA isoform function to generate novel association recommendations. mFRecSys uses a tri-factorization approach proposed by [53]. Matrix factorization (MF) occupies a critical role in recommendation systems. MF maps mRNA isoform and function terms from a user-item interaction matrix to a latent feature space. In this space, their dot product would predict mRNA isoform function associations. The tri-factorization approach used in mFRecSys allows incorporation of explicit biological context in predictions.

In mFRecSys, the “users” are the mRNA isoforms and the “items” are GO biological process terms. mRNA isoforms for *Mus musculus* were used in training mFRecSys. To develop a recommendation system to recommend mRNA isoform-specific functions, they characterized both mRNA isoforms and GO biological process terms.

mRNA isoform features include tissue-specific expression data, and sequence properties of mRNA and proteins. mRNA isoforms from the *Mus musculus* genome (GRCm38.p4 assembly) that have both mRNA and protein sequence were used. Tissue-specific expression features were extracted from 359 RNA seq samples from 17 mouse tissues from ENCODE [47]. Sequence features were extracted from mRNA and protein sequences (listed in Table 4.1 of [34]). Semantic similarity for all GO biological process terms was calculated. GO biological process terms were mapped to mRNA isoforms. These mappings were either positive, negative, or unknown. This is an important feature of mFRecSys. A gene-GO term association is “positive” if the gene is mapped to the GO term, “negative” if the gene is tagged with a “NOT” qualifier, and “unknown” for all other associations. If a gene produces single mRNA isoforms, and is mapped to a particular GO term, then this mRNA isoforms was considered positive for that term and its ancestor terms (“true path rule”). If a gene producing multiple mRNA isoforms is negative for a GO term, then all mRNA isoforms are considered negative for that GO term and all its child terms (inverse “true path rule”).

In their training sets, they included 75,826 mRNA isoforms and 18,869 GO biological process terms. A total of 6582 features were used to develop the recommendation system. Additionally, they developed 9 tissue-specific mFRecSys models using RNA-seq samples from the FANTOM5 project [54]. mFRecSys was validated using the latest annotations from GO. They found that a recommendation system model using only mRNA sequence properties (0.993) was better at mRNA isoform function prediction, while a model using only mRNA isoform expression (0.976) information performed the worst.

While mFRecSys tries to address some of the limitations of previous method, there is still room for improvement. More systems level properties such as explicit protein domains, PPIs, other OMICS data such as Ribo-Seq, proteomics, and metabolomics can be integrated. While the performance metrics reported in the original mFRecSys paper are very high, the authors do not perform a benchmark evaluation against previous methods. This makes it hard to assess the actual improvements (if any) of mFRecSys over previous methods. Covariates like age and sex were ignored.

#### 3.3.2. DisoFun

DisoFun [28] is another recommendation system based mRNA isoform function predictor. In DisoFun, a new collaborative matrix factorization technique is introduced that integrates PPI network data and GO hierarchy to differentiate between mRNA isoform specific functions. The goal of incorporating this information is to overcome the common assumption in other predictors, that the collected annotations of each gene are complete. However, the GO annotations of proteins are often incomplete [55]. While DeepIsoFun also uses GO hierarchy to train the model, it uses only expression information. DisoFun improves on previous predictors by incorporating PPI, GO hierarchy, and expression data to predict mRNA isoform specific functions.

DisoFun input includes mRNA isoform expression data, gene annotation data, PPI data and GO hierarchy information. For the mRNA isoform expression data, 298 samples were taken from the ENCODE project [47]. PPI data was collected from BioGRID [56]. The mRNA isoform expression data matrix and gene annotation data matrix were factorized into low-rank matrices. A low-rank matrix is shared between the two-resulting low-rank matrices to facilitate collaboration, along with the PPI data and GO hierarchy information. The functional associations between the mRNA isoforms and genes are encoded in a gene-isoform association matrix.

To assess the performance of DisoFun, the authors used a protocol referred to as “historical to recent”. Essentially, they trained DisoFun on human GO annotations from a historical version (2016) and validated the predictions on a later version (2018), accounting for structural changes in GO. They also tested the performance of various components of DisoFun, i.e., different variations of DisoFun that 1. Does not use GO hierarchy, 2. Does not use PPI data, 3. Does not use both, and 4. Uses both PPI and GO hierarchy. The authors report that the version of DisoFun that uses both components significantly outperforms the other versions, and that the contribution of GO hierarchy seems to be larger than that of PPI information.

The performance of DisoFun was also compared to the performance of other isoform function predictors- mi-SVM, WLRM, iMILP, DeepIsoFun, and IsoFun. For these comparisons, annotation information for human genes and isoforms (2018 version) was used. At both gene-level and mRNA isoform level function prediction comparisons, DisoFun outperformed the other methods for predicting GO biological process terms (AUROC: 0.71 vs 0.56–0.66). For mRNA isoform level predictions, 2782 single mRNA isoform human genes were used. DisoFun, IsoFun and mi-SVM were further tested on four multiple-isoform genes (*LMNA*, *ADAM15*, *BCL2L1*, and *CFLAR*), whose mRNA isoform level functions have been studied experimentally. The authors report that DisoFun had the highest prediction accuracy, compared to the other predictors.

Like DeepIsoFun and DIFFUSE, a 2-class classification system is used- a gene can be “positive” or “negative” with respect to a specific GO term. While comparing the performance of DisoFun at gene-level to other predictors, the authors of DisoFun selected twice as many negative genes as positive ones for each term for training the model, thereby potentially introducing bias in the model. While DisoFun introduces new information by using PPI data, it ignores the potential information carried in the mRNA isoform and protein sequences. Cell, tissue, or condition specific predictions are not made, which are a major aspect of mRNA isoforms. Age and sex specific functions are ignored. Like mFRecSys, DisoFun also makes novel mRNA isoform function predictions. The code for DisoFun can downloaded from their website (http://mlda.swu.edu.cn/codes.php?name=DisoFun).

## 4. Discussion

The completion of the human genome sequencing project resulted not only in a deeper understanding of the genomic complexities of humans, but also stressed the importance of alternative splicing in regulating phenotypic complexity [2]. The results from the human genome project revealed that most humans contain ~25,000 protein coding genes, which is remarkably close to the number of genes in a nematode, *C. elegans* (20,000 genes) and is less than that of rice (40,000 genes)—suggesting that organismal complexity cannot be explained merely by the number of genes. The concept of alternative splicing was consequently introduced to explain the phenotypic complexity of an organism. Alternative splicing led to an understanding that a gene can potentially encode multiple protein isoforms, each isoform capable of executing a unique function.

Despite the significant role of alternatively spliced mRNA isoforms in controlling organismal complexity, limited progress has been made towards annotating their functions. Limitations in experimental technologies have resulted in most large-scale genomic data to be profiled at gene level, rather than mRNA isoform level. Consequently, functions have been often assigned only at a gene level and the functions of the individual mRNA isoforms of a gene remain unannotated. However, recent developments in sequencing technology have resulted in several RNA-Seq datasets that provide information on mRNA isoform expression levels and an opportunity to understand the expression profile of genes at mRNA isoform levels. Such datasets also inspired the development of numerous computational methods as viable alternatives to experimental approaches for mRNA isoform function detection.

Computational approaches developed in the recent years have, to an extent, been able to address the shortcomings of experimental approaches (time and resource constraints) and have the capability to predict organism wide mRNA isoform functions. To start with, such approaches often require a gold standard dataset to train and test their prediction models. A gold standard dataset, in the context of mRNA isoform function prediction, can be expected to contain a set of genes and their mRNA isoforms with experimentally verified functions. However, the lack of such a dataset in the scientific community makes it difficult to train prediction models and the accuracy of predictions from computational methods becomes questionable. Therefore, most computational methods resort to using single mRNA isoform encoding genes and their annotations as the ground truth. One would then expect the predictions from these methods for multiple mRNA isoform encoding genes to be highly unreliable.

A summary of the computational methods reviewed in this work is summarized in Table 2. Most computational methods have been trained using datasets from either mouse or humans, limiting their applications to other species. It is known that alternatively spliced mRNA isoform sequences are often strongly dependent on the splicing mechanisms, which can vary by species. Therefore, a prediction method trained on human RNA-Seq datasets may learn the underlying functional annotation rules specific to human (and closely related species) mRNA isoform data and may not accurately predict the mRNA isoform functions in distinct species like plants, which observe a different splicing mechanism. The current state of the art computational methods could strongly benefit from using models that are trained on data from multiple species, which would make their predictions more robust and generic.

The field of mRNA isoform function prediction has developed rapidly since its inception. The need to annotate the functions of mRNA isoforms has become even more obvious in the recent years to understand their regulatory and functional behavior at the systems level. The field can greatly benefit by using multi-omics data within a computational framework to predict mRNA isoform functions. Integrative approaches combining protein structural data with multi-omics data could also be used to tune the performance of existing computational methods. In the coming years, the precise detection of mRNA isoform function is likely to encourage the use of mRNA isoforms as biomarkers for different diseases, rendering them as preferred therapeutic targets.

## Figures and Tables

**Table 1:** Frequently used terminologies and their contextual definitions in the field of mRNA isoform function prediction.

Terminology	Definitions
Alternative splicing	A transcriptional regulatory mechanism that leads to the production of multiple mature mRNA isoforms from a single gene.
mRNA isoforms	Mature mRNA products of the same gene which usually differ in their sequences and may perform different functions.
Multiple Instance Learning (MIL)	A weakly supervised learning framework where labels are available at the level of gene instead of the individual mRNA isoforms and the goal is to find the specific mRNA isoforms responsible for a gene’s function.
RNA-seq	A high-throughput way of measuring the expression of gene and mRNA isoforms.
Gene Ontology (GO) term	A controlled vocabulary term that refers to a specific function performed by genes and gene products.
mRNA isoform–isoform interaction (III) functional network	mRNA isoform level functional networks where an edge between two mRNA isoforms suggests the involvement of both mRNA isoforms in the same function.

**Table 2:** Summary of methods reviewed based on their input data type and approach.

Method	Method	Input Data Type	Input Data Description	Performance (GO Biological Process Terms)	Limitations
isoPred	MIL with support vector machine (SVM) as a base learner	RNA-seq; GO	19,209 genes and 24,274 mRNA isoforms from mouse	Area Under the Receiver Operating Curve (AUROC): 0.68–0.76 (multiple mRNA isoform genes) AUROC: 0.62–0.68 (single mRNA isoform genes)	Only RNA-Seq input; Random unannotated genes as negative set; no tissue, cell, sex, or age specificity
iMILP	MIL with label propagation	RNA-seq; GO	31,454 human mRNAs	AUROC: 0.67	Only RNA-Seq input; Genes annotated to sibling GO terms used as negative set; no tissue, cell, sex, or age specificity
IsoFunc	MIL with SVM as base learner	RNA-seq; GO	11,946 genes and 59,297 mRNA isoforms from human	AUROC: 0.64	Only RNA-Seq input; Random unannotated genes as negative set; no tissue, cell, sex, or age specificity
WLRM	MIL with weighted logistic regression	RNA-seq; GO	11,946 genes and 59,297 mRNA isoforms from human	AUROC: 0.6–0.85	Only RNA-Seq input; Random unannotated genes as negative set; no tissue, cell, sex, or age specificity
IIIDB	Network-based	RNA-seq; domain–domain interactions; GO; protein–protein interaction (PPI)	31,454 mRNA isoforms from human	Data not available	Only RNA-Seq input; Subcellular localization as negative set; no tissue, cell, sex, or age specificity; limited to existing PPIs
Mouse Splice Isoform Network	Network-based; MIL with Bayesian network	RNA-Seq; Exon array; Protein docking; pseudo-amino acid composition; GO; Pathways	Data not available	AUROC: 0.62	Random unannotated genes as negative set; no tissue, cell, sex, or age specificity
TENSION	Network- based; Random Forest	RNA-Seq; mRNA Sequence; Protein Sequence; PPI; GO; Pathways	21,813 genes and 75,826 mRNA isoforms from mouse	AUROC: 0.94	No cell, sex, or age specificity
DeepIsoFun	Deep learning	RNA-Seq; GO	19,532 genes and 47,393 mRNA isoforms from human	AUROC: 0.74	Only RNA-Seq input; Random unannotated genes as negative set; no tissue, cell, sex, or age specificity
DIFFUSE	Deep learning	RNA-Seq; mRNA sequence; Protein sequence; GO	19,303 genes and 39,375 mRNA isoforms from human	AUROC: 0.84	Random unannotated genes as negative set; no tissue, cell, sex, or age specificity
mFRecSys	Recommendation system	RNA-Seq; mRNA sequence; Protein sequence; PPI; GO; Pathways	21,813 genes and 75,826 mRNA isoforms from mouse	AUROC: 0.99	Limited tissue-specificity; No cell, sex, or age specificity
DisoFun	Recommendation system	RNA-Seq; PPI; GO	11,868 genes and 25,939 mRNA isoforms from human	AUROC: 0.71	Only RNA-Seq input; Random unannotated genes as negative set; no tissue, cell, sex, or age specificity

## Abbreviations

AS	Alternative Splicing
GO	Gene Ontology
KEGG	Kyoto Encyclopedia of Genes and Genomes
ML	Machine Learning
PPI	Protein-Protein Interaction
III	Isoform-Isoform Interaction
MIL	Multiple Instance Learning
PCC	Pearson Correlation Coefficient
AUROC	Area Under the Receiver Operating Characteristic
MD	Mean Difference
AUPRC	Area Under the Precision-Recall Curve
DA	Domain Adaptation
DNN	Deep Neural Network
CRF	Conditional Random Field
MF	Matrix Factorization
